# Double Hare-Lip

**Published:** 1871-02

**Authors:** 

**Affiliations:** University of Pennsylvania.


### ARTICLE VII.

### *Double Hare-lip.*

The case now before the class (a man 30 years of age), is that physical imperfection known as hare-lip. In this particular instance it differs, however, markedly from the others which it has come in my way to treat before you, in being a compound or double cleft.

In the usual hare-lip, as all here well know, the deficiency consists in a V break in the continuity of the part. The double break varies as markedly as the first is true to a type. The case before us is a fair example of what you will meet with in this direction of practice. It is not a complicated case neither it is a simple one. First you observe a break involving one-half, or much more nearly two-thirds, of the superior lip—an example of what is meant by deficient development. That nature, however, attempted, but was thwarted in her work of here making a perfect lip, is evidenced in that we find hanging from the septum of the nostrils a miss-shapen fleshy mass, the abortion, without doubt, of the lacking portion of the lip. This centre teat or part in these cases, is the pons asinorum, and it so various—so diversified in its relations to adjacent parts that the question of concern, in all such operations, is, what shall we do with it? Here, in the case before us, for example, is this teat so outside the harmonious line of the lip that a strip which should rest upon the cutaneous surface of either side, would pass beneath the mucous face of this middle piece.

As another example, I will here introduce a second patient, this infant, but three weeks of age. You have seldom seen anything more curious looking. It seems to have a fleshy door knob hanging from the tip of its nose. The child has scarcely the remnant of an upper lip, and what is a great deal worse, there is a complete break, both through the hard and soft palate, rendering the nose and mouth a common cavity, and which cavity has neither anterior or posterior closure, being, thus of course, always open. The babe cannot suckle; and when milk is poured into this cavity, attempt at deglutition throws about as much back as it gets down the œsophagus. Observe this case particularly and compare it with the first, since it enables me very well to show the two extremes of complicated hare lips. The pedunculated prominence hanging from the nose of this child is the rudimentary lip, and who shall say how much of the absent palate enters into its composition? All the inside ot the tumor is made up of bone; this the knife will be sure to demonstrate to us. The condition of this child is lamentable, and I shall exert myself to the utmost, you may be sure, for its relief.

In the meantime, until we come to the operation, consider it, each one of you his own patient, and devise in your own mind what kind of an operation you conceive best adapted to meet the requirements of the case.

From any operative standpoint, we divide the subject of double hare-lip into two classes—simple and complicated. Such a division answers our purpose very well.

Let us then for a moment refer to an uncomplicated double hare-lip where the centre piece or teat might be found so large and square as fairly to divide the lip into three parts; now here, the median line of the lip would be found in the center piece; it would, therefore, I think, suggest itself to any one that either side of the cleft was to be treated as a separate hare-lip—that is, the whole operation may be very well done at one sitting, but there would necessarily be symmetrical parings made of either cleft. In such a case we have also to take into account the concavity made on either of the sides of our fissures, as reference is had to the influence exerted on the free margin of the lip; for here, of course, we require no lateral made swell.

Whether again in these really double cases we would first operate on the one side, and when this was cured on the other, is a matter for the judgement of the operator. Many surgeons prefer to correct the whole deformity at one sitting. If he should do this, the operation would only deviate from the principles laid down, as regard would be had to the approximation of the parts. If the center-piece is small, I think it would be found the most satisfactory practice to pass the pins directly from one lateral flap to the other, on through the central teat, thus uniting all the parts together by a common suture. If, on the contrary, the center-piece is broad and well covered with skin, I think the greatest good is found in using two sets of sutures.

As regards the single or double operation, I myself am influenced by the width of the middle piece, the tenseness or laxity of the tissue of the lip, and the endurance and condition of the patient.

Another modification of the double hare-lip is one in which there is a projection into the cleft of the incisor teeth, the alveolar process being sufficiently normal to allow of non-interference with it. The projection of the teeth is a natural result of the lack of external support from the labial deficiency; the tongue has actually pushed them outwardly. This explanation will, I am sure, seem strange only to those who are acquainted with the exceeding mobility of the dental organs under slight but continued force. It is certainly the true cause of such projection. In a case of this kind, the preliminary operation is the removal of the teeth. If now six months are allowed to intervene before attempting the operation on the lip, the alveoli of the extracted teeth will be found to have receded through absorption quite the eighth of an inch. The second operation is then to be done, *secundem artem.* This waiting on the process of absorption will be found to conduce greatly to a successful result, but it is not a necessity.

A still better, though more tedious mode of correcting such a deformity, however, is by bringing the projecting teeth back to their normal place in the arch through the agency of elastic ligatures, which is a perfectly feasible operation, and not at all difficult of performance. By such a preliminary procedure we not only get the teeth out of the way, but we also save to the patient those valuable organs. To make and apply such a ligature, we have only to take a common slip of india-rubber; attach at either end a loop of silk; place the loops over certain of the molar teeth (it is immaterial which), and stretch the centre or rubber part across over the labial faces of the teeth to be pulled back. It is astonishing how quickly and powerfully such a force will act upon the teeth, in two or three weeks at most they will be brought into a proper line. To secure them in situ, and prevent their again being pushed forward, we have only to keep them ligatured in any convenient manner until the operation on the lip is made.

Cleft of the lip, as seen in the case of the babe before us, is found in almost every case of cleft of the hard palate. It has always been deemed very important in these cases that an operation on the lip should be performed as early as possible, since it is thought to favor closure of the bony cleft. In these cases the operation differs from that suited to an ordinary one, only when there is a projection of one or both alveolar processes into the break. In such instances, if the projection is very marked—that is, so much as to prevent the bringing of the lips together over it—we may, perhaps, be able to do nothing better than to cut away the parts. This however, is always to be avoided when possible: first, because we thus destroy the germs of the teeth; and secondly, because if by any means we can get union of the lip, the parts in their development will come mutually to accomodate each other. In such cases, some authors recommend that we endeavor to bend back these jttuings of bone, turning them in towards the mesial line; and when this can be done, it answers a very admirable purpose.

Still another mode consists in the employment of the fronto-occipito-labial elastic sling, which pulls upon the projecting process backward from the occiput. It will certainly fulfill the indications, but its application is not unattended with trouble.

The variations, together with all the anomalies in the direction of hare-lip are first to be studied, as regards their cure, from the artistic standpoint. The surgeon knows when and what he can afford to cut; he knows what nature will do in the case; it only remains for him to consider well his incision—where he shall make them, and what is to be the result; before the operation is attempted.

With this general view of the subject which will, I trust, be found by you sufficiently full for a satisfactory guidance in this direction of practice, we may turn to the performance of the operation upon these cases.

First, the man. It it a sad and unusual thing that he has been allowed to grow to adult age with such a deformity. As this central piece is not wide, we can utilize it and make a single operation suffice for both clefts; that is, we shall pare both edges of this central piece, then the edges of the lip upon either side, and passing long pins directly from the lateral flaps through this teat, bring all four raw surfaces into their proper positions by the single ligature thrown around the pins. In regard to the character of our incisions, it is not here so necessary to use the ellipse or double V parings which I spoke of when upon the subject of simple hare-lip, since we do not here produce in the same way the central median prominence and swell, and our object is, therefore, rather to produce perfectly fresh surfaces at every point that union may be rapid and complete.

[Operation performed as described, the teat being used to fill up the gap between the two flaps, and a portion of one of the parings being allowed to remain in order to add to the fullness of the free margin, somewhat after the manner of the operation of Mirault. But two pins were used, and adhesive strips were immediately applied to assist in support.—De F. W.]

In regard to this little infant, I trust you have all by this time decided as to the operation which you would perform were the case in your hands. The child is but three weeks old, yet in these cases of such terrible deformity the operation must be done early, since the compression effected even by the closure of the soft parts may at this tender age greatly influence also the bony cleft, for you well know the large proportion of animal matter which exists in the bones of young children, and the ease with which they may be bent. At some future clinic I shall dwell more fully upon the matter of cleft palate, and will not, therefore, now detain you.

I shall now proceed to remove this pendant mass from the septum by means of bone forceps, and, as you will then see, we have to deal with the lip as in ordinary cases of single hare-lip. The break is wide, but I think the tension will not be too great.

[The mass was removed and the lip pared with the double V incision ; long pins were used, and the parts easily drawn together by the usual suture, and assistance rendered by the adhesive strips. The mass proved to consist of bone (probably the rudimentary intermaxillary) and cartilage.—De F. W.]—*Dr. Garretson, University of Pennsylvania.*—*Medical and Surgical Reporter.*

